# Growth–maturity–egg production trade-offs and candidate gene associations with egg-laying persistency in slow-growing Thai native chickens

**DOI:** 10.14202/vetworld.2026.2685-2702

**Published:** 2026-07-06

**Authors:** Wootichai Kenchaiwong, Wuttigrai Boonkum, Jennarong Kammongkun, Khanitta Pengmeesri, Thassawan Somchan, Doungnapa Promket

**Affiliations:** 1 Faculty of Veterinary Science, Mahasarakham University, Mahasarakham 44000, Thailand.; 2 Department of Animal Science, Faculty of Agriculture, Khon Kaen University, Khon Kaen 40002, Thailand.; 3 Bureau of Animal Husbandry and Genetic Improvement, Department of Livestock Development, Pathum Thani 12000, Thailand.; 4 Branch of Animal Science, Department of Agricultural Technology, Faculty of Technology, Mahasarakham University, Mahasarakham 44150, Thailand.; 5 Branch of Animal Science, Department of Agricultural Technology, Faculty of Technology, Mahasarakham University, Mahasarakham 44150, Thailand.; 6 Branch of Animal Science, Department of Agricultural Technology, Faculty of Technology, Mahasarakham University, Mahasarakham 44150, Thailand.

**Keywords:** candidate genes, egg production, estimated breeding values, genetic correlations, indigenous poultry, principal component analysis, Thai native chickens, tropical adaptation

## Abstract

**Background and Aim:**

Slow-growing Thai native chickens are valuable genetic resources for sustainable poultry production under tropical conditions, but their reproductive efficiency is constrained by trade-offs among growth, sexual maturity, egg production, and egg-laying persistency. This study investigated the multivariate structure and genetic basis of these trade-offs and evaluated trait-specific associations between candidate gene polymorphisms and estimated breeding values (EBVs).

**Materials and Methods:**

Phenotypic, pedigree, and genotypic data were obtained from 563 female Pradu Hang Dum chickens across two generations. Growth performance, reproductive timing, egg production, and egg-laying persistency traits were recorded over a 365-day laying cycle. Principal component analysis (PCA) was used to characterize trait clustering. Genetic parameters and correlations were estimated using single- and bivariate animal models with average information restricted maximum likelihood procedures. Candidate gene polymorphisms in dopamine receptor D2 (*DRD2*), vasoactive intestinal peptide (*VIP*), melatonin receptor 1C (*MTNR1C*), and neuropeptide Y (*NPY*) were genotyped using polymerase chain reaction–restriction fragment length polymorphism and analyzed for associations with EBVs using linear mixed models.

**Results:**

PCA identified four principal components explaining 71.7% of the total phenotypic variance, clearly separating growth, sexual maturity, egg production output, and egg-laying persistency. Heritability estimates were high for birth weight (0.72), moderate for body weight at age at first egg (0.47) and persistency ratio (0.43), and low for cumulative egg number traits (0.08–0.12). Genetic correlations between egg number and persistency were strongly negative (−0.70 to −0.88). Candidate gene analyses detected significant genotype-dependent differences in EBVs for cumulative egg number at 360 days associated with *DRD2*, *VIP*, and *NPY* (p < 0.01), and *MTNR1C* (p < 0.05). Birds carrying the *VIP* DD genotype exhibited superior EBVs for egg number at 270 and 360 days, whereas the *DRD2* CC genotype showed significantly higher genetic merit for egg number at 360 days than the TT genotype. In contrast, the *NPY* SS and *MTNR1C* AA genotypes were associated with reduced egg production. No significant associations were detected between these candidate genes and growth or persistency traits.

**Conclusion:**

Egg-laying persistency represents a distinct biological dimension from growth and egg number in Thai native chickens. Incorporation of persistency into EBV-based multi-trait selection programs, together with the complementary use of candidate gene markers, may enhance long-term productivity and sustainability of indigenous chickens under tropical production systems. These findings provide a foundation for developing resilient dual-purpose breeding strategies that balance productivity and reproductive stability.

## INTRODUCTION

Indigenous chickens, typically characterized as slow-growing breeds, are increasingly recognized as valuable genetic resources for sustainable poultry production, particularly in tropical and low-input systems where tolerance to heat stress, endemic diseases, and variable management conditions is essential [1–3]. Thai native chickens represent such adaptive genetic resources, exhibiting high survival and strong environmental fitness under hot and humid conditions [4, 5]. Despite these adaptive advantages, Thai native chickens consistently exhibit lower reproductive efficiency than commercial layer lines, as reflected in moderate growth rates, delayed sexual maturity, and reduced egg production after peak lay [6, 7]. These limitations constrain overall productivity and hinder their wider adoption in sustainable and dual-purpose production systems. These performance patterns reflect inherent biological and genetic trade-offs among growth, reproduction, and environmental adaptation rather than management limitations alone [8, 9]. Selection strategies that emphasize rapid early growth or total egg number in isolation may disrupt physiological equilibrium and reduce long-term reproductive efficiency, particularly in slow-growing genotypes adapted to tropical environments [10, 11].

Egg-laying persistency is a key reproductive trait that reflects a hen's capacity to maintain stable egg production beyond peak lay [12, 13]. In contrast to cumulative egg number, persistency captures the temporal stability of laying performance and is closely associated with clutch length, pause duration, and neuroendocrine regulation [13, 14]. Evidence from commercial layer populations indicates that persistency is genetically and biologically distinct from total egg production, as high egg number does not necessarily translate into sustained laying performance over time [12, 14]. Recent studies investigating the genetic parameters of clutch length and hen-day egg production in Thai native chickens under heat stress have demonstrated that egg-laying persistency is an important trait to incorporate into breeding programs [15]. Moreover, most quantitative genetic studies in indigenous and slow-growing chickens have primarily emphasized growth traits and cumulative egg production, generally reporting low-to-moderate heritability estimates and, in some cases, unfavorable genetic correlations between early growth and reproductive performance [16, 17]. Although recent research has adopted multi-trait animal models and selection indices to enhance overall performance [6, 11], egg-laying persistency has rarely been explicitly evaluated, and its genetic relationships with growth and sexual maturity remain poorly quantified. Furthermore, while principal component analysis (PCA) has proven effective for resolving complex trait interrelationships and identifying biologically meaningful trait dimensions in poultry, its application to integrated analyses of growth, maturity, egg production, and persistency in Thai native chickens remains limited [18].

Candidate gene approaches continue to provide valuable insights into the biological mechanisms underlying complex reproductive traits, particularly those that are difficult or costly to measure phenotypically [19, 20]. Genes involved in neuroendocrine regulation, including dopamine receptor D2 (*DRD2*), vasoactive intestinal peptide (*VIP*), neuropeptide Y (*NPY*), and melatonin receptor 1C (*MTNR1C*), play central roles in appetite control, circadian rhythm, prolactin secretion, and oviposition behavior in birds [21–23]. Although polymorphisms in these genes have been associated with egg production traits in poultry [24–27], their associations with egg-laying persistency and EBV-based reproductive performance in slow-growing Thai native chickens remain largely unexplored. Nevertheless, recent evidence suggests that persistency-related traits, including clutch length and sustained laying performance under heat stress, are important indicators of long-term productivity and adaptation in tropical poultry populations [15].

Genes involved in neuroendocrine regulation play critical roles in controlling reproductive performance in poultry by modulating hormonal pathways that govern ovulation, feeding behavior, and circadian rhythms. *NPY* is an important neuromodulator influencing gonadal function, feed intake, and the secretion of key reproductive hormones, and has been associated with age at first egg (AFE) and egg production rate through its role in regulating ovulation [28]. Dopaminergic signaling also contributes to reproductive control, where dopamine receptor D2 (*DRD2*) inhibits prolactin secretion at the pituitary level, thereby reducing incubation behavior and promoting egg production; polymorphisms in *DRD2* have been linked to variation in egg production in Thai native chickens [29]. Similarly, *VIP* is a major stimulator of prolactin release in birds, and its gene expression and protein levels are closely associated with circulating prolactin concentrations, with several polymorphisms reported to affect total egg number in chickens [23]. In addition, melatonin and its receptor *MTNR1C*, which is unique to avian species, play important roles in regulating circadian rhythms and reproductive physiology, with evidence linking melatonin receptor polymorphisms to AFE and reproductive traits through endocrine pathways involving estradiol and gonadotropin-inhibitory hormone [22].

Estimated breeding values (EBVs) derived from mixed model genetic evaluations provide robust measures of additive genetic merit by integrating phenotypic, pedigree, and environmental information [6, 11]. However, few studies have jointly examined EBVs and candidate gene polymorphisms within a multivariate framework that explicitly accounts for growth–maturity–egg production trade-offs. Previous studies in Thai native chickens have mainly focused on cumulative egg production, test-day records, or heat stress responses, while candidate genes such as *NPY* and *MTNR1C* have generally been evaluated individually for their effects on egg number. Consequently, the biological relationships among growth, sexual maturity, total egg output, and egg-laying persistency remain incompletely understood. Moreover, no previous study has simultaneously applied multivariate PCA to distinguish these traits as separate biological dimensions and integrated this approach with animal-model genetic analyses and EBV-based associations involving neuroendocrine candidate genes (*DRD2*, *VIP*, *NPY*, and *MTNR1C*) throughout an entire 365-day laying cycle in a slow-growing Thai native chicken population. Addressing these knowledge gaps is essential for developing dual-purpose breeding strategies to improve long-term productivity, reproductive stability, and adaptation to tropical low-input production systems.

Therefore, the present study aimed to investigate the multivariate relationships among growth performance, sexual maturity, egg production, and egg-laying persistency using PCA and to estimate the genetic parameters and correlations among these traits in slow-growing Thai native chickens. In addition, the study evaluated the associations between polymorphisms in the neuroendocrine candidate genes *DRD2*, *VIP*, *NPY*, and *MTNR1C* and EBVs for growth, reproductive, and persistency traits. By integrating multivariate phenotypic analyses, animal-model genetic evaluation, and candidate–gene associations, this study provides a biologically informed framework for sustainable breeding strategies and supports the development of resilient dual-purpose Thai native chicken lines optimized for productivity and environmental adaptation in tropical production systems.

## MATERIALS AND METHODS

### Ethical approval

All procedures involving animals were reviewed and approved by the Institutional Animal Care and Use Committee (IACUC) of Mahasarakham University, Mahasarakham, Thailand (Approval No. IACUC-MSU-026-016/2025). The study was conducted in accordance with institutional guidelines for the ethical care and use of animals in research and complied with the principles of the 3Rs (Replacement, Reduction, and Refinement). All procedures were designed to minimize animal discomfort and distress, and sample collection and animal handling were performed by trained personnel in accordance with accepted veterinary and animal welfare practices.

### Study period and location

The study was conducted from January 2018 to November 2020 using Pradu Hang Dum Thai native chickens maintained at the Chiang Mai Livestock Research and Breeding Center, Chiang Mai, Thailand. The production system represented a tropical semi-intensive environment typical of sustainability-oriented native chicken breeding programs. Ambient temperatures ranged from 21.3°C to 32.5°C, with an average relative humidity of approximately 52%.

### Study design

A longitudinal, population-based genetic study was conducted on 563 female Pradu Hang Dum chickens from two consecutive generations. Phenotypic, pedigree, and genotypic information was integrated to investigate relationships among growth performance, sexual maturity, egg production, and egg-laying persistency. Multivariate analyses, genetic parameter estimation, and candidate–gene association analyses were performed to characterize growth–maturity–egg production trade-offs and to evaluate marker effects on EBVs.

### Animals and management

Animals used in this study were obtained from the Chiang Mai Livestock Research and Breeding Center (Chiang Mai, Thailand). A total of 563 female Pradu Hang Dum chickens from two generations (2018–2020) were included. This population structure under tropical semi-intensive management was considered representative of sustainability-oriented native chicken breeding systems.

The breeding population was maintained as a closed nucleus population with within-breed selection, in which approximately 30 cocks and 150 hens were selected in each generation (effective population size ≈100). Each cock was mated with five hens through artificial insemination, and mating plans were designed to minimize inbreeding. Pedigree records comprised 5,676 individuals. Approximately 2,500 chicks were produced per generation, from which approximately 40 cocks and 270 hens were retained for performance testing. Hens exhibiting poor laying performance or failure to lay were removed before selecting the top 30 cocks and 150 hens for the subsequent generation.

Birds were individually identified using wing bands and reared in open-sided houses with natural ventilation. After a 21-day incubation period, chicks were stocked at a density of 8 birds/m² until 18 weeks of age, then transferred to individual battery cages.

Birds were fed according to age. From hatch to 4 weeks, chicks received a starter diet containing 21% crude protein (CP) and 3,000 kcal/kg metabolizable energy (ME). From 4 to 12 weeks, a grower diet containing 19% CP and 2,900 kcal/kg ME was provided. Feed and water were supplied ad libitum throughout the experimental period.

Continuous lighting (24 h light:0 h dark) using 100-W heating lamps was applied from hatch to 4 weeks, followed by natural daylight from 4 to 12 weeks of age. After 18 weeks of age, birds were maintained under ambient tropical conditions. At 20 weeks of age, hens were transferred to individual cages (20 × 45 × 40 cm; floor area ≈0.09 m²) and remained individually housed throughout the laying period. All birds received the same commercial layer diet (19% CP and 2,900 kcal ME/kg) at 110 g/day with free access to water. A consistent 12 h light/day schedule was maintained during the laying period.

All birds were managed in accordance with Good Agricultural Practices, and vaccination schedules were implemented by the farm veterinarian in accordance with Thai Agricultural Standards. Under semi-intensive management, Thai native chickens typically attain market weights of 1.2-1.4 kg at 12-14 weeks of age, with feed conversion ratios of 3.0-3.5, survival rates exceeding 90%, and carcass yields ranging from 68% to 72%.

### Data collection and measurements

**Growth performance traits:** Growth performance was evaluated using standardized procedures. Individual body weights were recorded using calibrated scales at hatch (BW0) and at 4, 8, 12, and 16 weeks of age (BW4, BW8, BW12, and BW16, respectively). All measurements were recorded individually to ensure accuracy and reliability.

**Egg production traits:** Seven egg production-related traits were recorded, including body weight at first egg (AFE_WT), AFE, egg weight at first egg (AFE_EW), egg weight at 270 days (EW270), egg weight at 360 days (EW360), cumulative egg number at 270 days (EN270), and cumulative egg number at 360 days (EN360). Measurements were obtained over a complete 365-day laying cycle. Unequal sample sizes across traits resulted from incomplete records and were addressed using mixed-model analyses.

**Egg-laying persistency traits:** Because repeated longitudinal egg production records over the entire laying cycle were unavailable, simplified persistency indicators based on late-stage egg production were used instead of model-based persistency measures.

The persistency ratio (PR) was defined as


\[
PR=\frac{{R}_{late}}{{R}_{early}}
\]

Where

\({R}_{early}=\frac{E{N}_{early}}{\left(270,AFE\right)}\)and \({R}_{late}=\frac{E{N}_{late}}{90}\)

Persistency late share (PLS) was calculated as


\[
PLS=\frac{E{N}_{late}}{E{N}_{360}}=\frac{E{N}_{late}}{E{N}_{early}+E{N}_{late}}
\]

where \(E{N}_{early}\)represents the number of eggs produced from AFE to 270 days of age and \(E{N}_{late}\)represents the number of eggs produced between 271 and 360 days of age. Therefore, the early laying period was standardized according to individual AFE. These simplified indices were considered appropriate for indigenous chickens, which characteristically exhibit clutch-based laying patterns with prolonged but gradually declining production under tropical conditions.

### DNA extraction and candidate gene genotyping

Blood samples (1 mL) were collected from the wing vein into 1.5-mL microcentrifuge tubes containing 0.5 M EDTA. Genomic DNA was extracted from whole blood using the guanidine hydrochloride method [30].

Polymorphisms in the candidate genes *DRD2*, *VIP*, *MTNR1C*, and *NPY* were analyzed using polymerase chain reaction–restriction fragment length polymorphism (PCR-RFLP). Gene-specific primers were used to amplify *DRD2* (chromosome 24; GenBank accession number 428252), *VIP* (chromosome 3; GenBank accession number 396323), *MTNR1C* (chromosome 4; GenBank accession number JQ249896), and *NPY* (chromosome 2; GenBank accession number M87298).

**Figure 1:**
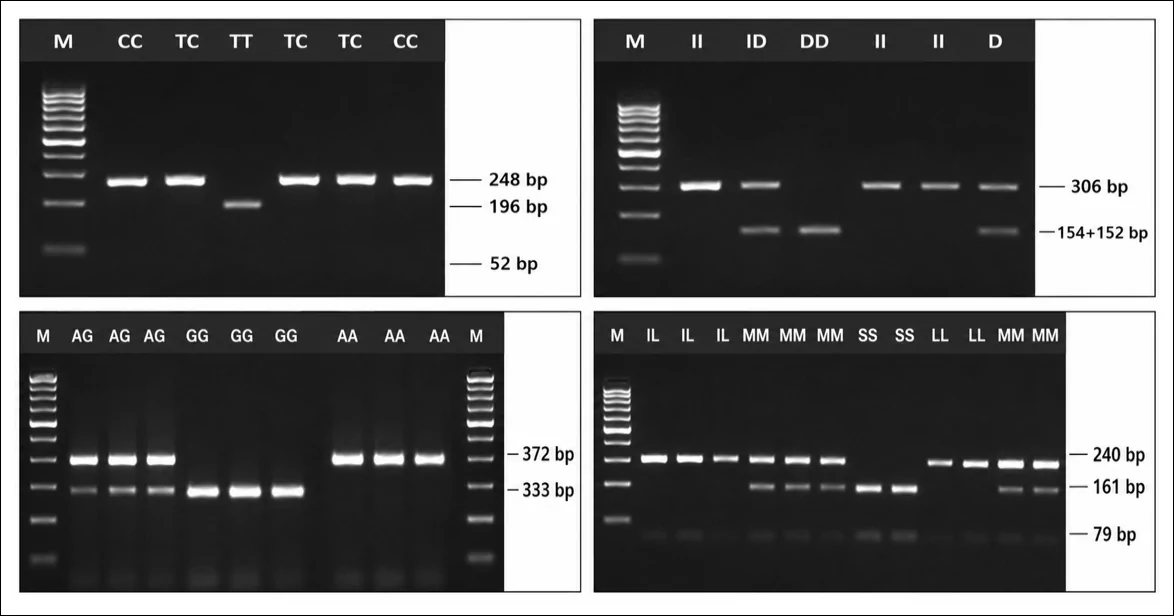
Genotyping patterns of candidate gene polymorphisms associated with growth and reproductive traits in Thai native chickens using polymerase chain reaction–restriction fragment length polymorphism analysis. (A) *DRD2* digested with *Bse*GI; M = 100 bp DNA ladder; TT genotype (196 and 52 bp), TC genotype (248, 196, and 52 bp), and CC genotype (248 bp). (B) *VIP* digested with *Vsp*I; II genotype (306 bp), ID genotype (306, 154, and 152 bp), and DD genotype (154 and 152 bp). (C) *MTNR1C* digested with *Mbo*I; AA genotype (372 bp), AG genotype (372 and 333 bp), and GG genotype (333 bp). (D) *NPY* digested with *Dra*I; LL genotype (240 bp), MM genotype (240, 161, and 79 bp), and SS genotype (161 and 79 bp).

PCR amplification was performed using annealing temperatures of 60°C for *DRD2* and *NPY* and 58°C for *VIP* and *MTNR1C*. Amplified fragments were digested with *Bse*GI, *Vsp*I, *Mbo*I, and *Dra*I, respectively, and separated on 2.5% agarose gels. Genotypes were assigned according to restriction fragment patterns [22, 23] (Figure 1). Samples with ambiguous banding patterns were excluded. To confirm genotyping accuracy, a subset of samples was randomly re-genotyped. All polymorphisms investigated in this study have previously been reported as synonymous or non-coding variants [22, 23].

### Statistical analysis

**PCA:** Trait clustering was evaluated using a PCA-derived path diagram to visualize relationships among principal components and phenotypic performance traits at the individual bird level. The analysis was performed using JASP statistical software version 0.95.4 (JASP Team). All phenotypic variables were standardized before analysis, and rotated component structures were obtained using the oblique via cluster method to improve interpretability. Complete rotated component loadings and communalities are provided in Supplementary Table-S1. This approach facilitated the identification of biological and physiological relationships among trait groups.

Subsequently, PCA was performed using combined genotype data from *DRD2*, *VIP*, *MTNR1C*, and *NPY* to characterize the multivariate genetic structure underlying variation in individual performance. Unlike previous applications of PCA to egg production stages in commercial layers or egg quality traits, the present study incorporated growth, sexual maturity, and two distinct persistency indicators (PR and PLS) to resolve biological trade-offs in a slow-growing indigenous population.

Genotype and allele frequencies, polymorphism information content, and expected heterozygosity were calculated as described by Falconer and Mackay [31]. Conformity with Hardy–Weinberg equilibrium was evaluated using the chi-square (χ²) test.

**Genetic parameter estimation:** Contemporary group, defined by hatch and generation, was included as a fixed effect for growth traits and AFE in Model 1. For egg production traits, including AFE_WT, AFE_EW, egg weights at 270 and 360 days (EW270 and EW360), egg numbers at 270 and 360 days (EN270 and EN360), and persistency traits (PR and PLS), AFE was included as a covariate in Model 2.

Variance components and heritability estimates were obtained using single-trait animal models, whereas genetic correlations were estimated using bivariate linear animal models. Variance components were estimated using the average information restricted maximum likelihood algorithm implemented in the BLUPF90 program [32]. Heritability estimates were obtained using single-trait animal models, and genetic correlations were estimated using bivariate analyses. Convergence was assumed when changes in log-likelihood values between successive iterations were <10⁻¹².

EBVs derived from single-trait models were subsequently used in candidate gene association analyses.

The bivariate animal model is expressed as:


\[
\begin{aligned}
{y}_{1}={X}_{1}{\beta }_{1}+{Z}_{1}{a}_{1}+{\varepsilon }_{1} \\
{y}_{2}={X}_{2}{\beta }_{2}+{Z}_{2}{a}_{2}+{\varepsilon }_{2}
\end{aligned}
\]

where y₁ and y₂ are vectors of observations for traits 1 and 2, respectively; β represents the vector of fixed effects; a denotes additive genetic effects; and **ε** represents residual effects. X and Z are incidence matrices relating observations to fixed and random effects, respectively. Additive genetic effects were assumed to follow:


\[
a∼N(0,A{\sigma }_{a}^{2})
\]

where A is the additive relationship matrix.

Heritability (h²) was calculated as:


\[
{h}^{2}=\frac{{\sigma }_{a}^{2}}{{\sigma }_{a}^{2}+{\sigma }_{e}^{2}}
\]

where σ²a is additive genetic variance and σ²e is residual variance.

Genetic (rg) and residual (re) correlations between traits were estimated as:


\[
\begin{aligned}
{r}_{g}=\frac{{\sigma }_{a12}}{\sqrt{{\sigma }_{a1}^{2}{\sigma }_{a2}^{2}}} \\
{r}_{e}=\frac{{\sigma }_{e12}}{\sqrt{{\sigma }_{e1}^{2}{\sigma }_{e2}^{2}}}
\end{aligned}
\]

where σa12 and σe12 represent additive genetic and residual covariances between traits, respectively.

**Candidate gene association analysis:** Associations between candidate gene polymorphisms and EBVs were evaluated using a linear mixed model. Genotype was fitted as a fixed effect for growth traits (BW0, BW4, BW8, BW12, and BW16) and reproductive traits (AFE, AFE_WT, AFE_EW, EW270, EW360, EN270, and EN360). Analyses were performed using SAS software version 9.4 [33].

The model was expressed as:


\[
{y}_{ijk}=\mu +{Gene}_{j}+{\varepsilon }_{ijk}
\]

where \({y}_{ijk}\)represents the EBV for trait *i*, μ is the overall mean, Genej is the fixed effect of genotype *j*, and εijk is the random residual error.

No additional covariates were included because EBVs had already been adjusted for pedigree structure and fixed environmental effects through animal-model analyses. Consequently, using EBVs rather than raw phenotypic values enabled candidate gene associations to be evaluated while accounting for pedigree relationships and environmental influences.

## RESULTS

### Summary statistics of growth performance and egg production traits

The descriptive statistics for body weight, egg production, and egg persistency traits are presented in Table-1. The mean BW0 of Thai indigenous chickens was 29.10 ± 3.35 g, with moderate variability (%CV = 11.51). Body weight increased progressively with age, reaching 255.23 ± 50.93 g at BW4, 735.36 ± 95.37 g at BW8, 1160.35 ± 113.65 g at BW12, and 1464.68 ± 127.97 g at BW16. The %CV declined with advancing age, from 19.96 at BW4 to 8.74 at BW16, indicating reduced relative variability in body weight as birds matured. Considerable phenotypic ranges were observed for all growth traits, particularly at later ages, with BW16 ranging from 1100 to 2182 g.

The mean AFE_WT was 2031.90 ± 210.78 g, while the mean AFE was 162.80 ± 15.17 days. AFE_EW averaged 36.06 ± 6.32 g, showing relatively high variability (%CV = 17.52). EN270 averaged 154.53 ± 28.76 eggs and increased to 181.46 ± 31.78 eggs at EN360. Both traits exhibited moderate-to-high variability, with %CV values of 18.61 and 17.52, respectively, and wide ranges across individuals. EW270 averaged 46.18 ± 4.39 g and increased slightly to 46.77 ± 4.82 g at EW360, with relatively low variability compared with egg number traits.

PR averaged 0.24 ± 0.24, with a %CV of 100.00 and values ranging from 0.00 to 1.82. Similarly, persistency of laying score (PLS) averaged 0.15 ± 0.11, with a high %CV of 72.64 and a maximum value of 0.58. These results indicate substantial individual variation in laying persistency during the later production period.

**Table 1:** Descriptive statistics of growth performance, egg production, and egg persistency traits in Thai indigenous chickens.

**Traits**	**N**	**Mean**	**SD**	**%CV**	**Min**	**Max**
Growth performance						
BW0 (g)	507	29.10	3.35	11.51	19.00	40.00
BW4 (g)	507	255.23	50.93	19.96	115.00	406.00
BW8 (g)	507	735.36	95.37	12.97	443.00	1004.00
BW12 (g)	490	1160.35	113.65	9.79	796.00	1616.00
BW16 (g)	484	1464.68	127.97	8.74	1100.00	2182.00
Egg production						
AFE_WT (g)	545	2031.90	210.78	10.37	1075.00	2652.00
AFE (d)	545	162.80	15.17	9.32	130.00	211.00
AFE_EW (g)	545	36.06	6.32	17.52	21.00	64.00
EN270 (egg)	545	154.53	28.76	18.61	53.00	221.00
EN360 (egg)	545	181.46	31.78	17.52	83.00	266.00
EW270 (g)	427	46.18	4.39	9.52	32.00	59.00
EW360 (g)	545	46.77	4.82	10.30	31.00	62.00
Egg persistency						
ENearly	545	153.97	28.31	18.39	64.00	221.00
ENlate	545	27.45	20.77	75.67	0.00	110.00
PR	545	0.24	0.24	100.00	0.00	1.82
PLS	545	0.15	0.11	72.64	0.00	0.58

BW0 = birth weight, BW4 = body weight at 4 weeks, BW8 = body weight at 8 weeks, BW12 = body weight at 12 weeks, BW16 = body weight at 16 weeks, AFE_WT = body weight at age at first egg, AFE = age at first egg, AFE_EW = egg weight at first egg, EN270 = cumulative egg number at 270 days of age, EN360 = cumulative egg number at 360 days of age, EW270 = egg weight at 270 days of age, EW360 = egg weight at 360 days of age, ENearly = number of eggs produced from AFE to 270 days of age, ENlate = number of eggs produced from 271 to 360 days of age, PR = persistency ratio, PLS = persistency late share, SD = standard deviation, %CV = coefficient of variation.

### PCA and trait clustering

**Figure 2:**
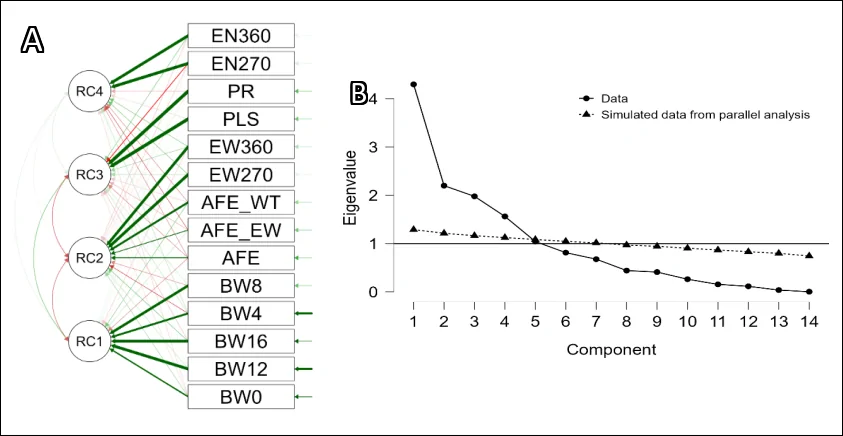
(A) PCA correlation biplot network showing variable loadings of the first four rotated components. Green and red lines indicate positive and negative loadings, respectively, and line thickness reflects loading magnitude. The first rotated component is associated with growth performance traits (BW0–BW16), the second with sexual maturity and egg weight traits (AFE, AFE_WT, AFE_EW, EW270, and EW360), the third with egg persistency traits (PR and PLS), and the fourth with cumulative egg production traits (EN270 and EN360). (B) Scree plot of eigenvalues for phenotypic traits. Based on the elbow rule and Kaiser criterion (eigenvalue >1), four components were retained.

PCA was performed to evaluate multivariate relationships among growth performance, reproductive timing, egg production, and egg persistency traits (Figure-2). Bartlett’s test of sphericity indicated that the data were suitable for PCA (χ² = 6465.336, df = 91, p < 0.001). Based on an inspection of the scree plot and PC-based parallel analysis, four PCs were retained, explaining 71.7% of the total phenotypic variance. This multivariate structure highlights distinct biological clustering among growth, maturity, egg production, and persistency traits that have been less clearly characterized in Thai native chicken populations.

PC1 explained 22.6% of the variance and showed high loadings for body weight traits measured from hatch to 16 weeks of age (BW0–BW16). PC2 accounted for 19.6% of the variance and was primarily associated with AFE and the corresponding body weight and egg weight traits. PC3 explained 16.0% of the variance and was characterized by high loadings for egg number and egg weight traits at later stages of production. PC4 accounted for 13.4% of the variance and exhibited strong loadings for persistency-related traits (PR and PLS), along with a positive association with EN270. The PCA loading pattern indicated clear clustering of growth performance, maturity, egg production, and egg persistency traits across distinct components (Figure 2).

**Figure 3:**
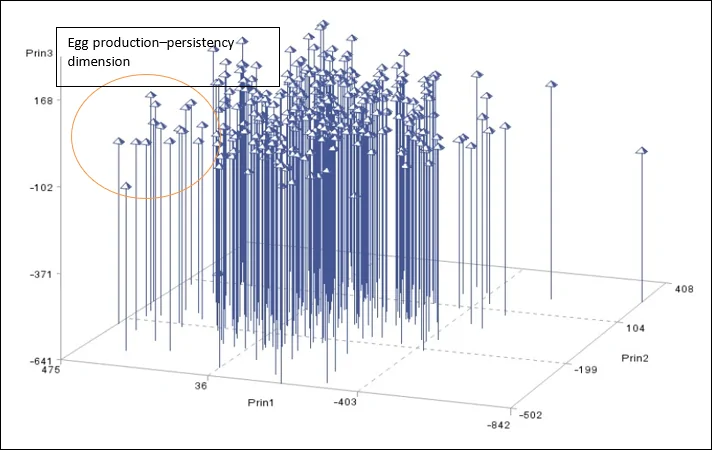
Three-dimensional PCA score plot showing the distribution of observations along Prin1 (growth), Prin2 (maturity), and Prin3 (egg production and persistency). The circled region indicates observations with high Prin3 scores, representing favorable egg-laying persistency and cumulative egg production. Axis dispersion suggests partial independence among growth, maturity, and egg production dimensions.

The three-dimensional PCA plot (Figure-3) showed separation of individuals along the Prin3 axis, corresponding primarily to egg production and persistency traits. Individuals with higher Prin3 scores exhibited higher values for persistency-related traits and cumulative egg production, whereas variation along Prin1 and Prin2 reflected differences in growth and maturity-related traits. A limited number of individuals displayed favorable scores across all three principal axes.

### Effects of candidate gene polymorphisms on trait clustering

**Figure 4:**
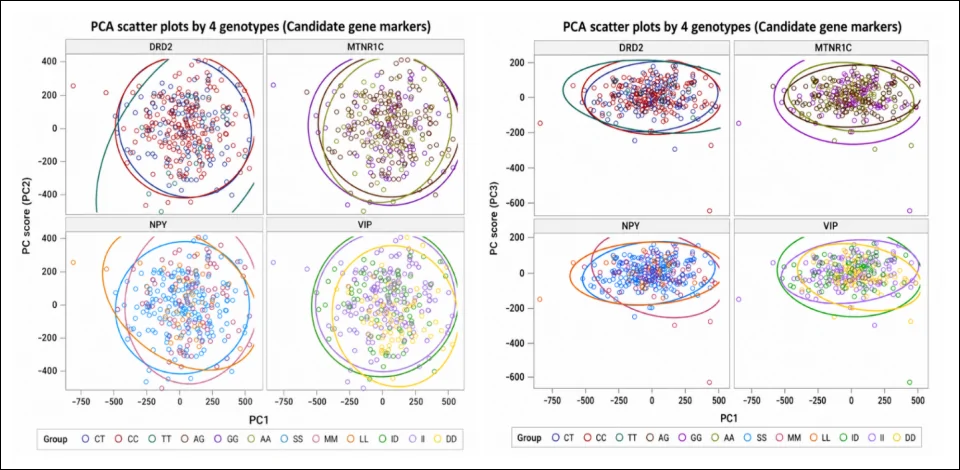
PCA scatter plots showing the distribution of individuals across (A) PC1 and PC2 and (B) PC1 and PC3 based on all phenotypic traits, with individuals grouped by *DRD2*, *MTNR1C*, *NPY*, and *VIP* genotypes. Extensive overlap among genotype groups indicates that genotype-associated variation is small relative to total phenotypic variation across the PCs.

**Figure 5:**
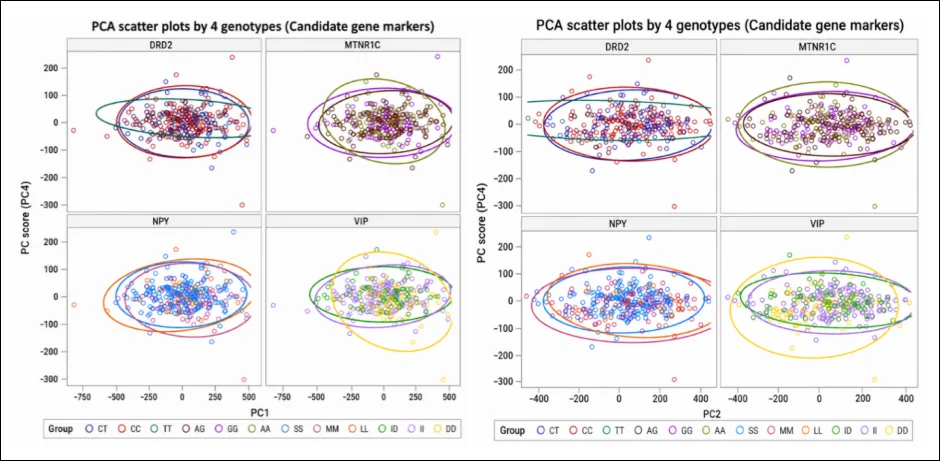
PCA scatter plots showing the distribution of individuals across (A) PC1 and PC4 and (B) PC2 and PC4 based on all phenotypic traits, with individuals grouped by *DRD2*, *MTNR1C*, *NPY*, and *VIP* genotypes. Extensive overlap among genotype groups indicates that genotype-associated variation is small relative to total phenotypic variation across the PCs.

**Figure 6:**
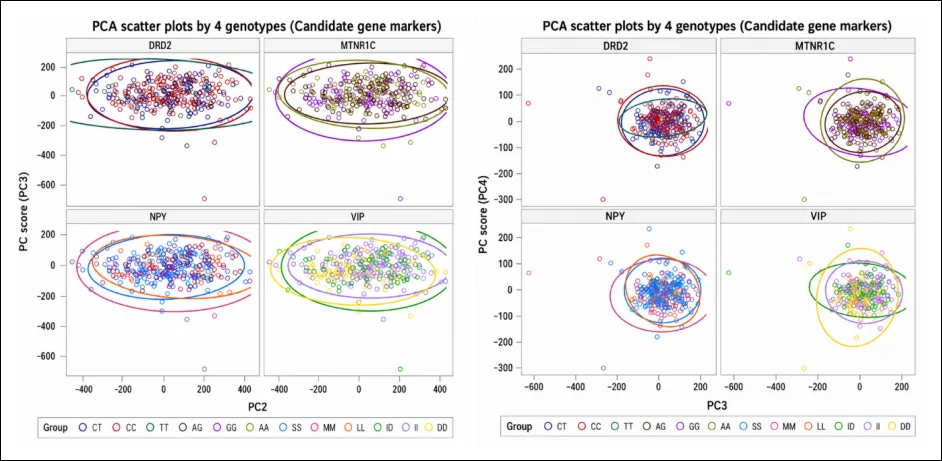
PCA scatter plots showing the distribution of individuals across (A) PC2 and PC3 and (B) PC3 and PC4 based on all phenotypic traits, with individuals grouped by *DRD2*, *MTNR1C*, *NPY*, and *VIP* genotypes. Extensive overlap among genotype groups indicates that genotype-associated variation is small relative to total phenotypic variation across the PCs.

Figures 4–6 present PCA plots of all phenotypic traits, with individuals grouped by *DRD2*, *MTNR1C*, *NPY*, and *VIP* genotypes. Across all PCA planes (PC1–PC4), genotype classes showed wide dispersion and substantial overlap, with no distinct genotype-specific clustering or separation. Similar distribution patterns were observed for growth, sexual maturity, cumulative egg number, and egg persistency traits. Persistency-related components displayed variation largely independent of growth- and maturity-associated components across genotypic groups.

Overall, PCA patterns did not indicate discrete multivariate phenotypic profiles associated with any single polymorphism. The extensive overlap among genotype groups suggests that individual candidate genes had relatively small effects on overall phenotypic variation in Thai native chickens.

Table 2 presents the genotype and allele frequencies, polymorphism information content, and Hardy–Weinberg equilibrium test results for candidate genes in Thai native chickens. The allele frequencies of *MTNR1C* were relatively balanced between alleles. Polymorphism information content values for *DRD2*, *VIP*, *MTNR1C*, and *NPY* were moderate, ranging from 0.33 to 0.38, indicating moderate levels of genetic polymorphism within the population. In addition, the *DRD2* and *MTNR1C* loci conformed to Hardy–Weinberg equilibrium (χ² < 3.84). In contrast, the *VIP* locus exhibited a strong deviation from equilibrium (χ² = 119.23) despite showing moderate polymorphism. The *NPY* locus showed a high frequency of the S allele (0.71) and deviation from Hardy–Weinberg equilibrium (χ² = 7.26).

### Genetic parameter estimates

Genetic parameter estimates derived from models incorporating candidate gene marker information are presented in Table 3. For growth performance traits, heritability estimates ranged from moderate-to-high, with the highest estimate observed for BW0 (h² = 0.72 ± 0.09). Post-hatch body weights exhibited moderate heritability, including BW4 (0.24 ± 0.08), BW8 (0.35 ± 0.10), BW12 (0.44 ± 0.10), and BW16 (0.35 ± 0.08).

For egg production traits, AFE_WT showed moderate heritability (0.47 ± 0.10), whereas AFE and AFE_EW exhibited lower heritability estimates of 0.22 ± 0.07 and 0.07 ± 0.05, respectively. Cumulative egg number showed low heritability at EN270 (0.08 ± 0.03) and EN360 (0.12 ± 0.05). Egg weight at later production stages exhibited low-to-moderate heritability, with estimates of 0.23 ± 0.08 for EW270 and 0.10 ± 0.07 for EW360.

Egg persistency traits exhibited low-to-moderate heritability estimates, with values of 0.43 ± 0.11 for PR and 0.20 ± 0.09 for PLS. The moderate heritability estimated for PR represents a potentially useful finding for persistency-related selection in Thai native chickens. Across most egg production traits, residual variance exceeded additive genetic variance, whereas a greater proportion of additive genetic variance was observed for early growth and persistency-related traits.

### Genetic and residual correlations among traits

Genetic and residual correlations among growth performance traits, age and weight at first egg, egg production traits, and egg persistency traits are presented in Table 4. Genetic correlations among body weight traits measured at different ages (BW0–BW16) were positive and ranged from low to high magnitude (0.15–0.67).

**Table 2:** Genotype and allele frequencies, polymorphism information content, and Hardy–Weinberg equilibrium test results for candidate genes in Thai native chickens.

**Gene**	**Genotype frequencies**			**Allele frequencies**		**χ²**	**PIC**	**He**	**p-value**
DRD2	CC = 0.41	CT = 0.47	TT = 0.12	C = 0.65	T = 0.36	0.55	0.35	0.45	0.866
VIP	II = 0.50	ID = 0.24	DD = 0.26	I = 0.62	D = 0.38	119.23	0.36	0.47	<0.001
MTNR1C	AA = 0.25	AG = 0.49	GG = 0.26	A = 0.50	G = 0.51	0.42	0.38	0.50	0.899
NPY	LL = 0.11	MM = 0.36	SS = 0.53	L = 0.29	S = 0.71	7.26	0.33	0.41	0.172

χ² = Chi-square value for Hardy–Weinberg equilibrium test, PIC = Polymorphism information content, He = Expected heterozygosity, DRD2 = Dopamine receptor D2, VIP = Vasoactive intestinal peptide, MTNR1C = Melatonin receptor 1C, NPY = Neuropeptide Y. The p-values indicate deviation from Hardy–Weinberg equilibrium. Values are expressed as frequencies.

**Table 3:** Genetic parameter estimates derived from integrated candidate gene marker data.

**Traits**	**Additive variance**	**Residual variance**	**Heritability (h²)**	**SE**
Growth performance				
BW0 (g)	8.0	3.1	0.72	0.09
BW4 (g)	280.1	898.3	0.24	0.08
BW8 (g)	1743.0	3205.9	0.35	0.10
BW12 (g)	4556.1	5686.5	0.44	0.10
BW16 (g)	5499.4	10141.0	0.35	0.08
Egg production				
AFE_WT (g)	16196.0	18066.0	0.47	0.10
AFE (d)	29.7	107.9	0.22	0.07
AFE_EW (g)	2.3	30.6	0.07	0.05
EN270 (egg)	70.1	780.6	0.08	0.03
EN360 (egg)	113.7	627.6	0.12	0.05
EW270 (g)	2.8	9.4	0.23	0.08
EW360 (g)	1.3	12.0	0.10	0.07
Egg persistency				
PR	207.6	278.9	0.43	0.11
PLS	17.9	70.7	0.20	0.09

BW0 = birth weight, BW4 = body weight at 4 weeks, BW8 = body weight at 8 weeks, BW12 = body weight at 12 weeks, BW16 = body weight at 16 weeks, AFE_WT = body weight at age at first egg, AFE = age at first egg, AFE_EW = egg weight at first egg, EN270 = cumulative egg number at 270 days of age, EN360 = cumulative egg number at 360 days of age, EW270 = egg weight at 270 days of age, EW360 = egg weight at 360 days of age, PR = persistency ratio, PLS = persistency late share, SE = standard error.

Early body weight traits showed positive genetic correlations with AFE, with estimates of 0.47 for BW4 and 0.22 for BW8. In contrast, BW4 and BW8 exhibited negative genetic correlations with EN360, ranging from −0.23 to −0.43.

Egg persistency traits showed negative genetic correlations with body weight from 4 to 16 weeks of age (BW4–BW16). Genetic correlations between BW4–BW16 and PR ranged from −0.54 to −0.39, whereas those with PLS ranged from −0.36 to −0.33. EN270 and EN360 also showed strong negative genetic correlations with egg persistency traits. Genetic correlations between EN270 and PR and PLS were −0.88 and −0.70, respectively, whereas corresponding correlations between EN360 and PR and PLS were −0.82 and −0.87. These strong negative genetic correlations indicated unfavorable relationships between cumulative egg number and persistency traits in slow-growing Thai native chickens.

Residual correlations between EN360 and egg persistency traits were positive, ranging from 0.57 to 0.80. Residual correlations among growth, egg production, and persistency traits generally differed in magnitude and direction from their corresponding genetic correlations.

**Table 4:** Pairwise genetic and residual correlations among growth performance traits (BW0–BW16), body weight and age at first egg (AFE_WT and AFE), egg weight at first egg (AFE_EW), cumulative egg number (EN270 and EN360), egg weight at later production stages (EW270 and EW360), persistency ratio (PR), and persistency late share (PLS).

**Traits**	**BW0**	**BW4**	**BW8**	**BW12**	**BW16**	**AFE_WT**	**AFE**	**AFE_EW**	**EW270**	**EN270**	**EW360**	**EN360**	**PR**	**PLS**
BW0	—	0.39	0.38	0.26	0.54	0.19	−0.13	−0.15	−0.11	0.05	−0.21	−0.06	0.20	0.52
BW4	0.35	—	0.48	0.67	0.55	0.35	0.47	−0.40	0.28	0.08	0.05	−0.23	−0.39	−0.33
BW8	0.55	0.76	—	0.15	0.42	−0.31	0.22	−0.52	−0.12	−0.14	−0.24	−0.43	−0.51	−0.35
BW12	0.38	0.35	0.77	—	0.36	0.75	0.01	0.05	0.28	0.23	0.18	0.22	−0.50	−0.35
BW16	0.46	0.41	0.53	0.64	—	0.21	−0.08	−0.52	0.24	0.48	0.23	0.05	−0.54	−0.36
AFE_WT	0.22	0.20	0.21	0.15	0.27	—	−0.06	0.17	0.17	0.40	0.15	0.57	0.25	0.20
AFE	0.21	−0.31	−0.03	0.03	0.03	−0.73	—	−0.20	0.17	−0.20	0.01	−0.35	0.25	0.29
AFE_EW	0.53	0.17	0.20	0.05	0.18	0.24	−0.05	—	0.28	−0.49	0.32	−0.07	0.64	−0.01
EW270	0.44	0.00	0.11	0.15	0.10	−0.15	0.47	−0.06	—	−0.09	0.92	−0.29	0.06	0.06
EN270	−0.21	0.12	0.00	−0.12	−0.01	−0.30	0.36	−0.05	0.04	—	−0.03	0.80	−0.88	−0.70
EW360	0.44	0.09	0.14	0.09	−0.01	−0.08	0.37	−0.06	0.85	0.00	—	−0.17	−0.07	−0.07
EN360	−0.03	0.29	0.06	−0.08	0.03	−0.06	0.05	−0.18	0.00	0.71	−0.04	—	−0.82	−0.87
PR	−0.02	0.03	0.06	0.09	0.07	0.03	−0.20	−0.75	0.00	−0.12	0.38	0.80	—	0.92
PLS	0.00	0.38	0.26	0.30	0.30	0.16	0.16	0.00	0.00	0.09	0.09	0.57	0.92	—

Genetic correlations are shown above the diagonal, and residual correlations are shown below the diagonal. BW0, BW4, BW8, BW12, and BW16 = Body weight at hatch and at 4, 8, 12, and 16 weeks of age, respectively, AFE_WT = Body weight at first egg, AFE = Age at first egg, AFE_EW = Egg weight at first egg, EW270 and EW360 = Average egg weight at 270 and 360 days of age, respectively, EN270 and EN360 = Egg number up to 270 and 360 days of age, respectively, PR = Persistency ratio, PLS = Persistency of laying score. Negative values indicate inverse relationships between traits.

### Associations between candidate genes and traits

Table 5 presents the associations of *DRD2* and *VIP* polymorphisms with EBVs for growth performance, egg production, and persistency traits in Thai native chickens. Polymorphisms in *DRD2* and *VIP* were significantly associated with EBVs for EN270 and EN360 (p < 0.05).

Table 6 presents the associations of *NPY* and *MTNR1C* polymorphisms with EBVs for growth performance, egg production, and persistency traits in Thai native chickens. Polymorphisms in *NPY* and *MTNR1C* were significantly associated with EBVs for EN360 (p < 0.05).

Chickens carrying the *DRD2* CC genotype showed significantly higher EBVs for EN360 than those with the TT genotype (1.61 vs. −4.14). Similarly, the *VIP* DD genotype was associated with superior egg production performance compared with the II genotype, particularly for EN360 (2.84 vs. −0.64) and EN270 (4.22 vs. −1.10). In contrast, the *NPY* SS and *MTNR1C* AA genotypes were associated with lower egg production performance. However, no significant associations were detected between these candidate genes and growth or laying persistency traits.

## DISCUSSION

The present study provides original insights by demonstrating, for the first time in Thai native chickens, that egg-laying persistency constitutes a partially independent biological and genetic dimension, separable from growth, sexual maturity, and total egg output, as revealed by PCA and bivariate animal models. This finding has important implications for sustainable breeding because most existing genetic evaluations in Pradu Hang Dum and similar breeds have prioritized cumulative egg number or heat stress resilience using reaction norms or random regression, often overlooking long-term laying stability.

### Growth performance and egg production traits

The growth pattern of Thai native chickens observed in this study reflects a slow, steady growth rate, a defining characteristic of indigenous chickens raised under tropical conditions [4, 5]. This slow-growing phenotype represents an adaptive strategy that prioritizes heat tolerance and metabolic efficiency rather than rapid tissue accretion [34, 35]. Reproductive traits further reinforce the indigenous characteristics of this population, as the observed AFE (approximately 163 days) and AFE_WT (approximately 2032 g) are comparable to previous reports in Thai native chickens, which typically reach sexual maturity later than commercial layers because of slower physiological and endocrine development [2, 36]. The gradual increase in egg weight with advancing laying age reflects normal maturation of the reproductive system in indigenous chickens [7, 17]. A decline in egg-laying persistency during the late production phase is also characteristic of indigenous chickens and low-input production systems [12]. The high coefficient of variation for PR and the strong genetic correlations with egg number traits should be interpreted with caution because ratio-based persistency traits may be affected by part–whole dependency and denominator effects [37].

**Table 5:** Association of DRD2 and VIP polymorphisms with EBVs for growth performance, egg production, and persistency traits in Thai native chickens.

**Traits**	**Genotypes of ** ***DRD2*** ** gene**			**p-value**	**Genotypes of ** ***VIP*** ** gene**			**p-value**
	**CC**	**CT**	**TT**		**DD**	**ID**	**II**	
BW0	0.08 ± 0.16	0.17 ± 0.15	−0.09 ± 0.31	0.74	0.09 ± 0.21	0.10 ± 0.21	0.11 ± 0.15	0.99
BW4	0.20 ± 0.54	0.82 ± 0.51	−0.31 ± 1.02	0.52	0.51 ± 0.69	0.23 ± 0.71	0.48 ± 0.49	0.95
BW8	0.72 ± 2.03	3.11 ± 1.92	−0.98 ± 3.86	0.53	2.02 ± 2.60	0.68 ± 2.69	1.91 ± 1.85	0.92
BW12	1.52 ± 3.46	4.63 ± 3.27	−1.00 ± 6.58	0.68	3.15 ± 4.43	0.99 ± 4.57	3.27 ± 3.14	0.91
BW16	1.71 ± 3.98	5.72 ± 3.76	−1.91 ± 7.56	0.59	3.64 ± 5.09	1.65 ± 5.26	3.65 ± 3.62	0.95
AFE	−0.35 ± 0.22	0.05 ± 0.21	0.13 ± 0.41	0.34	−0.47 ± 0.28	0.04 ± 0.29	0.01 ± 0.20	0.32
AFE_EW	0.00 ± 0.03	−0.01 ± 0.03	0.07 ± 0.05	0.37	0.02 ± 0.04	−0.04 ± 0.04	0.02 ± 0.03	0.33
AFE_WT	1.03 ± 5.10	−1.41 ± 4.81	4.66 ± 5.67	0.33	4.72 ± 6.50	−5.54 ± 6.72	5.02 ± 4.62	0.17
EN270	0.15 ± 1.21ᵃ	1.76 ± 1.14ᵃ	−5.09 ± 2.29ᵇ	0.03	4.22 ± 1.54ᵃ	−0.92 ± 1.59ᵇ	−1.10 ± 1.09ᵇ	0.01
EN360	1.61 ± 0.35ᵃ	0.52 ± 0.33ᵇ	−4.14 ± 0.67ᶜ	<0.0001	2.84 ± 0.46ᵃ	0.17 ± 0.47ᵇ	−0.64 ± 0.32ᵇ	<0.0001
EW270	−0.03 ± 0.06	0.05 ± 0.06	0.08 ± 0.12	0.58	0.06 ± 0.08	−0.09 ± 0.09	0.05 ± 0.06	0.34
EW360	−0.02 ± 0.05	0.04 ± 0.04	0.06 ± 0.09	0.57	0.05 ± 0.06	−0.07 ± 0.06	0.03 ± 0.04	0.34
PLS	0.19 ± 0.13	−0.16 ± 0.12	−0.29 ± 0.25	0.09	−0.06 ± 0.17	0.01 ± 0.17	−0.04 ± 0.12	0.96
PR	0.88 ± 0.61	−0.89 ± 0.58	−1.14 ± 1.17	0.08	−0.52 ± 0.79	0.15 ± 0.82	−0.16 ± 0.56	0.83

Data are presented as least squares means ± SE. EBV = Estimated breeding value, SE = Standard error, BW0, BW4, BW8, BW12, and BW16 = Body weight at hatch and at 4, 8, 12, and 16 weeks of age, respectively, AFE = Age at first egg, AFE_EW = Egg weight at first egg, AFE_WT = Body weight at first egg, EN270 and EN360 = Egg number up to 270 and 360 days of age, respectively, EW270 and EW360 = Average egg weight at 270 and 360 days of age, respectively, PLS = Persistency of laying score, PR = Persistency ratio. Means within the same row and gene with different superscripts differ significantly (p < 0.05).

**Table 6:** Association of NPY and MTNR1C polymorphisms with EBVs for growth performance, egg production, and persistency traits in Thai native chickens.

**Traits**	**Genotypes of ** ***NPY*** ** gene**			**p-value**	**Genotypes of ** ***MTNR1C*** ** gene**			**p-value**
	**LL**	**MM**	**SS**		**AA**	**AG**	**GG**	
BW0	0.30 ± 0.32	0.26 ± 0.17	−0.05 ± 0.14	0.32	0.13 ± 0.21	0.11 ± 0.15	0.06 ± 0.20	0.97
BW4	1.33 ± 1.06	1.16 ± 0.58	−0.24 ± 0.48	0.12	0.45 ± 0.70	0.50 ± 0.50	0.28 ± 0.68	0.96
BW8	5.32 ± 3.99	4.59 ± 2.18	−1.10 ± 1.79	0.08	1.62 ± 2.63	2.04 ± 1.88	0.93 ± 2.55	0.94
BW12	5.32 ± 3.99	4.59 ± 2.18	−1.09 ± 1.79	0.06	2.48 ± 4.48	3.72 ± 3.21	0.99 ± 4.34	0.88
BW16	9.45 ± 6.78	8.00 ± 3.70	−2.29 ± 3.05	0.09	3.23 ± 5.15	3.95 ± 3.69	1.67 ± 4.99	0.93
AFE	−0.49 ± 0.43	−0.11 ± 0.23	−0.03 ± 0.19	0.629	0.08 ± 0.28	−0.21 ± 0.20	−0.10 ± 0.27	0.70
AFE_EW	−0.09 ± 0.06	0.03 ± 0.03	0.01 ± 0.02	0.16	−0.01 ± 0.04	0.02 ± 0.03	0.00 ± 0.04	0.76
AFE_WT	1.75 ± 5.02	8.30 ± 5.47	−3.23 ± 4.51	0.27	−3.55 ± 6.60	3.72 ± 4.72	2.02 ± 6.40	0.67
EN270	1.79 ± 2.38	1.76 ± 1.30	−0.99 ± 1.07	0.22	1.26 ± 1.57	−0.51 ± 1.12	0.89 ± 1.52	0.59
EN360	1.98 ± 0.72^a^	1.37 ± 0.39^a^	−0.51 ± 0.32^b^	<0.0001	−0.24 ± 0.48^a^	0.29 ± 0.34^ab^	1.33 ± 0.46^b^	0.04
EW270	0.11 ± 0.13	−0.04 ± 0.07	0.04 ± 0.06	0.51	0.05 ± 0.08	0.04 ± 0.06	−0.05 ± 0.08	0.63
EW360	0.08 ± 0.09	−0.03 ± 0.05	0.03 ± 0.04	0.51	0.04 ± 0.06	0.03 ± 0.04	−0.04 ± 0.06	0.62
PLS	−0.03 ± 0.26	0.04 ± 0.14	−0.08 ± 0.12	0.80	−0.24 ± 0.17	0.08 ± 0.12	−0.03 ± 0.17	0.31
PR	0.21 ± 1.22	−0.09 ± 0.66	−0.31 ± 0.55	0.91	−1.33 ± 0.80	0.28 ± 0.57	0.06 ± 0.77	0.25

Values are expressed as estimated breeding values (EBV) ± standard error (SE). Means within the same row and gene with different superscripts (a,b) differ significantly (p < 0.05). BW0, BW4, BW8, BW12, and BW16 = Body weight at hatch and at 4, 8, 12, and 16 weeks of age, respectively, AFE = Age at first egg, AFE_EW = Egg weight at first egg, AFE_WT = Body weight at first egg, EN270 and EN360 = Egg number up to 270 and 360 days of age, respectively, EW270 and EW360 = Average egg weight at 270 and 360 days of age, respectively, PLS = Persistency of laying score, PR = Persistency ratio. Gene names *NPY* and *MTNR1C* should be presented in italics.

### PCA and trait clustering

The characterization based on principal components indicates that growth, sexual maturity, cumulative egg production, and egg-laying persistency represent partially independent biological dimensions rather than a single productivity continuum. The first principal component consistently captured overall growth patterns in early body weight traits, suggesting that somatic growth constitutes a stable, genetically structured dimension largely distinct from reproductive timing and egg-laying performance. This pattern is consistent with previous quantitative genetic studies reporting limited overlap between growth-related traits and egg production or persistency [9, 38].

Previous multivariate analyses have shown that early and cumulative egg production load on separate principal components, with early production reflecting initial laying intensity and later components capturing variation associated with persistency across the laying cycle [10]. Similarly, Venturini *et al.* [39] reported that total egg output and post-peak production decline are represented by different principal components, indicating that egg number and egg-laying persistency can be statistically distinguished despite moderate correlations. More recent studies further suggest that persistency loads independently from body weight and production rate, reflecting potential trade-offs between sustained laying capacity and other economically important traits [18].

In the present study, the three-dimensional PCA score plot revealed a latent phenotypic pattern in which favorable persistency and higher cumulative egg production were distributed along a distinct production–persistency axis, while substantial dispersion remained across growth and maturity axes. This multiaxial structure is consistent with longitudinal and genomic evidence identifying egg-laying persistency as a distinct dimension contributing to late-cycle productivity and adaptability [14, 40, 41]. Together, these results support consideration of egg-laying persistency as a complementary trait to total egg number in breeding programs targeting long-term production stability. For example, incorporating persistency-related traits into multi-trait selection indices may help identify hens with more stable long-term laying performance rather than selecting solely for early cumulative egg number.

### Effects of candidate gene polymorphisms on trait clustering

The PCA genotype scatter plots showed substantial overlap among genotypes for all candidate genes, indicating that growth, egg production, and egg-laying persistency are not dominated by single major loci. This pattern is consistent with a polygenic genetic architecture for economically important traits, as reported in previous longitudinal and quantitative genetic studies [40]. The limited genotype-specific separation observed in PCA space suggests that polymorphisms in *DRD2*, *VIP*, *NPY*, and *MTNR1C* exert modest, trait-dependent effects rather than defining distinct multivariate phenotypic profiles.

The absence of distinct genotype clustering may reflect the highly polygenic nature of these traits and the limited ability of PCA to detect subtle effects of individual loci relative to total phenotypic variation. This observation is consistent with genome-wide association studies showing that reproductive traits are typically influenced by numerous loci with small effects [42]. Despite the absence of clear phenotypic clustering, *DRD2* and *MTNR1C* exhibited moderate polymorphism and balanced allele frequencies (Table-2), indicating that these loci retain genetic variation within the population. In contrast, deviations from the Hardy–Weinberg equilibrium observed for *VIP* and *NPY* may reflect population structure or historical selection pressure. These loci have been implicated in neuroendocrine pathways associated with reproductive regulation, suggesting potential biological relevance [13]. Overall, the present results are consistent with recent genomic studies indicating that candidate genes are more appropriately considered within multivariate or genomic selection frameworks rather than as single-gene predictors of performance [14].

### Genetic parameter estimates

The heritability estimates in this study are largely consistent with previous reports in native and tropical chicken populations. The very high heritability for BW0 (h² = 0.72) is slightly higher than most published estimates for Thai native and indigenous chickens but follows the same pattern of stronger genetic determination at hatch and early life [6, 43]. As birds aged, heritability of body weight declined to moderate levels (0.24–0.35), which is consistent with estimates reported for Thai native, Korean native, Iranian Mazandaran, and Ethiopian Tilili chickens [7, 11, 16]. This reduction likely reflects increasing environmental and management influences and is particularly relevant under tropical conditions, where growth rate is constrained by heat stress and resource availability. Differences in inheritance rates across ages may result from increased environmental variation with advancing age [43, 44].

In Thai native chickens, Boonkum *et al.* [34] demonstrated that heat stress slows growth while maintaining moderate additive genetic variance, supporting the present observation that later growth traits are more strongly environmentally modulated. AFE_WT showed moderate heritability (h² = 0.47), consistent with estimates reported in Thai purebred and hybrid layers [45], supporting its potential relevance as a selection criterion. In contrast, the low heritability estimates for egg number (0.08–0.12) closely match those reported in both native and commercial chickens reared under tropical environments [10, 45]. Egg weight exhibited low-to-moderate heritability, consistent with reports indicating relatively stable genetic control across breeds [17]. Importantly, the heritability of PR (h² = 0.43) falls within the moderate range reported for persistency or post-peak stability in commercial layers, whereas the lower heritability of PLS suggests greater environmental sensitivity during the late laying period [12, 40].

Genetic correlations revealed biologically relevant trade-offs that distinguish Thai native chickens from intensively selected commercial layers. Positive genetic correlations between early body weight and AFE indicate that genetically heavier birds tend to mature later, consistent with previous findings in both Thai native and commercial populations [16, 45]. In contrast, egg persistency traits showed consistently negative genetic correlations with growth traits and cumulative egg number, indicating that alleles favoring rapid growth or high total egg output tend to be associated with reduced long-term laying stability. The strong negative genetic correlations between egg number traits (EN270 and EN360) and persistency traits (PR and PLS) demonstrate that, in Thai native chickens, high egg number does not necessarily correspond to stable or persistent egg production.

This pattern contrasts with many studies in commercial layers, where persistency and egg number are often favorably correlated under controlled environments [12, 40]. Previous studies using random regression and spline-based approaches in commercial and Thai native chickens generally reported moderate-to-high positive genetic correlations between egg number and persistency-related traits, particularly during post-peak production stages [46]. This contrast is consistent with emerging evidence that egg-laying persistency represents a distinct biological dimension linked to metabolic resilience and reproductive longevity rather than cumulative egg output alone [14]. Nevertheless, the positive residual correlations between egg number and persistency observed in this study suggest that improvements in nutrition, housing, and health management may simultaneously enhance both traits at the phenotypic level. Collectively, these results emphasize that sustainable genetic improvement in Thai native chickens may benefit from multi-trait selection indices that explicitly incorporate egg-laying persistency alongside egg number and growth traits, in combination with management strategies that mitigate environmental constraints such as heat stress [34, 45].

### Candidate genes for selection in Thai native chickens

The candidate genes evaluated in this study (*DRD2*, *VIP*, *NPY*, and *MTNR1C*) were selected because they are known regulators of neuroendocrine pathways controlling reproduction, energy balance, and circadian rhythms in poultry. Ngu *et al.* [23] demonstrated that *VIP* plays a central role in regulating prolactin secretion and reproductive activity in birds, whereas Tu *et al.* [21] reported that dopaminergic signaling through *DRD2* exerts inhibitory control over prolactin release. Ngu *et al.* [23] further showed that prolactin-related endocrine regulation primarily influences laying performance rather than somatic growth.

In the present study, no consistent associations were detected between polymorphisms in *DRD2*, *VIP*, *NPY*, or *MTNR1C* and EBVs for growth traits, AFE, or egg weight, indicating that these genes are unlikely to represent major genetic determinants of growth or sexual maturity. This pattern is consistent with the findings of Fu *et al.* [47], who reported in a genome-wide association study that growth and egg weight traits are highly polygenic and influenced by numerous loci of small effect rather than by single candidate genes.

In contrast, significant genotype-dependent differences were observed for EBVs of cumulative egg number, particularly EN360, suggesting a trait-specific contribution of the investigated genes to long-term egg production. The present findings for *MTNR1C* and *NPY* are consistent with previous studies reporting significant associations of neuroendocrine-related genes with cumulative egg production traits in chickens [26, 48]. Similar associations between melatonin receptor gene polymorphisms and egg production traits have also been reported in chickens and ducks [24, 25, 47]. Collectively, these findings support the role of *DRD2*, *VIP*, *NPY*, and *MTNR1C* as supplementary molecular markers for improving long-term egg yield in breeding programs. Because EBVs are shrinkage-based estimates derived from mixed models, candidate gene associations should be interpreted cautiously, as part of the observed variation may depend on the genetic evaluation model.

Although persistency definitions are simple ratios, their integration with PCA and candidate genes provides a practical framework readily applicable to other tropical indigenous breeds. These findings suggest that incorporating persistency-related EBVs into multi-trait selection strategies may help balance long-term productivity and resilience in indigenous chicken breeding programs. Several limitations should be considered when interpreting the present findings. Egg number traits showed relatively low heritability, and tropical environmental conditions may have increased environmental variance affecting reproductive performance. In addition, candidate gene associations were based on EBVs derived from mixed models, while deviations from Hardy–Weinberg equilibrium at the *VIP* and *NPY* loci may reflect selection history or population structure. Furthermore, the functional effects of the studied polymorphisms were not experimentally validated.

## CONCLUSION

This study demonstrated that egg-laying persistency is a partially independent biological and genetic dimension in Thai native chickens, distinct from growth, sexual maturity, and cumulative egg production. PCA separated growth, maturity, egg production, and persistency traits into different components, while genetic analyses showed moderate heritability for BW0, AFE_WT, and PR, but low heritability for cumulative egg number traits. Strong negative genetic correlations between EN270, EN360, and persistency traits indicated that higher cumulative egg production does not necessarily reflect stable long-term laying performance in this population.

From a practical perspective, these findings suggest that breeding programs for Thai native chickens should not rely solely on cumulative egg number or early growth traits. Instead, multi-trait EBV-based selection indices should include egg-laying persistency to improve long-term reproductive stability while maintaining productivity under tropical conditions. Candidate gene polymorphisms in *DRD2*, *VIP*, *NPY*, and *MTNR1C* were associated mainly with cumulative egg number, particularly EN360, suggesting that these markers may serve as supplementary tools for improving long-term egg yield rather than as primary predictors of persistency.

The strength of this study lies in its integrated approach, combining phenotypic trait clustering, animal-model genetic parameter estimation, and EBV-based candidate gene association analysis over a 365-day laying cycle in a slow-growing indigenous chicken population. However, the findings should be interpreted with caution because persistency traits were based on simplified ratio-derived indicators, egg number traits showed low heritability, and deviations from Hardy–Weinberg equilibrium were observed for the *VIP* and *NPY* loci. In addition, the functional effects of the investigated polymorphisms were not experimentally validated.

Future studies should use longitudinal egg production records, larger multi-generation populations, genomic selection approaches, and functional validation of candidate variants to clarify the biological mechanisms underlying laying persistency. Overall, incorporating egg-laying persistency with cumulative egg production and candidate gene information may support the development of resilient, productive, and sustainable Thai native chicken lines for tropical low-input production systems.

## DATA AVAILABILITY

The datasets generated during the current study are available upon reasonable request from the corresponding author. There were no deviations from the approved experimental protocol during the study.

## GENERATIVE AI DECLARATION

The authors declare that generative artificial intelligence (AI) tools were used solely to improve language, grammar, and readability during manuscript preparation. All scientific content, data analysis, interpretation of results, and conclusions were developed and verified by the authors. The authors take full responsibility for the accuracy, integrity, and originality of the work presented, and no AI tool was listed as an author.

## AUTHORS’ CONTRIBUTIONS

WK: Conceptualization, study design, data collection, data analysis, interpretation of the results, and drafting and revision of the manuscript. WB: Interpretation of the results and drafting and revision of the manuscript. JK, KP, and TS: Data collection and manuscript review and revision. DP: Conceptualization, study design, data collection, data analysis, interpretation of the results, drafting and revision of the manuscript, and supervision of the study. All authors have read and approved the final version of the manuscript.
